# Oncology Clinicians and the Minnesota Medical Cannabis Program: A Survey on Medical Cannabis Practice Patterns, Barriers to Enrollment, and Educational Needs

**DOI:** 10.1089/can.2018.0029

**Published:** 2018-10-01

**Authors:** Dylan Zylla, Grant Steele, Justin Eklund, Jeanne Mettner, Tom Arneson

**Affiliations:** ^1^ Park Nicollet Oncology Research, HealthPartners Institute, Frauenshuh Cancer Center, Minneapolis, Minnesota.; ^2^ HealthPartners Institute, HealthPartners, Minneapolis, Minnesota.; ^3^ Minnesota Department of Health, St. Paul, Minnesota.

**Keywords:** cancer, cannabis, clinician survey, education, marijuana, research

## Abstract

**Background:** Medical cannabis has been available in the State of Minnesota since July 2015 through the Minnesota Medical Cannabis Program (MMCP).

**Objectives:** Our study aimed to delineate oncology providers' views on medical cannabis, identify barriers to patient enrollment, and assess clinicians' interest in a clinical trial of medical cannabis in patients with stage IV cancer.

**Methods:** From June to August 2017, we distributed a 14-question survey to Minnesota oncology physicians, advanced practice nurses, and physician assistants who care for adults and children with cancer. Descriptive analyses for each question were provided for all survey respondents.

**Results:** Of the 529 eligible survey participants, 153 (29%) responded to our survey; 68 respondents were registered with the MMCP. Most identified themselves as a medical oncologist or medical oncology nurse practitioner/physician assistant (*n*=125, 82%), and most practiced in a community setting (*n*=102, 67%). Overall, 65% of respondents supported the use of medical cannabis. Perceived cost and inadequate research were the highest barriers to MMCP patient enrollment. The lowest barriers included lack of health group support for allowing certification of patients and risk of social stigma. Of all respondents, 36% lacked confidence in discussing the risks and benefits of medical cannabis, and 85% wanted more education.

**Conclusions:** Although support for cannabis use in the cancer setting is growing, significant barriers remain. This study illustrates a clear need to give clinicians both data and education to guide their discussions about the benefits, risks, and cost considerations of using medical cannabis for cancer-related symptoms.

## Introduction

Cannabis use is becoming more widespread in the United States; currently, 9 states and the District of Columbia have legalized the use of cannabis for both recreational and medical purposes, with an additional 22 states legalizing for medical purposes only.^1^ Patients with cancer often have symptoms such as pain, nausea/vomiting, and anorexia, for which cannabis may provide benefit.

Medical cannabis has been available in the State of Minnesota since July 2015 through the Minnesota Medical Cannabis Program (MMCP). Any clinician (physician, advanced practice registered nurse [APRN], or physician assistant [PA]) may register with MMCP and can then certify patients with cannabis-eligible diagnoses for which they actively manage. Eligible diagnoses currently include epilepsy, cancer, intractable pain, HIV/AIDS, and many others. Clinicians get no formal training and make no specific recommendations about cannabis type/dosing. Certified patients then meet with trained pharmacists at one of the state's two registered cannabis manufacturers to purchase cannabis products. From July 2015 to December 2017, 1532 Minnesota patients were certified for medical cannabis use for cancer-related symptoms. Most cancer patients are certified by clinicians in the oncology field, including^2^

- medical oncologists (33%);
- nurse practitioners (NPs) or PAs (23%); and
- pain or palliative care specialists (19%).

Primary care providers certify ∼15% of all cancer patients. As such, the views and practice patterns of oncology clinicians are important drivers of cannabis use in patients with cancer.

Elsewhere in the nation, researchers have been working to more clearly delineate the use of medical cannabis in oncology patients. A recent survey of cancer patients at a large comprehensive cancer center in Seattle, Washington, showed that 21% of respondents reported using cannabis in the past month for physical symptoms (e.g., pain, nausea, and appetite loss) or neuropsychiatric symptoms (e.g., stress, depression, and insomnia).^3^ This survey also showed that most patients surveyed (74%) wanted information about cannabis from their cancer care team, yet just fewer than 15% reported receiving it.

Potential risks and benefits of cannabis use in the cancer setting have been reviewed,^4–9^ but robust randomized trials are lacking. Many factors appear to limit the use of medical cannabis in the cancer population, including (1) the lack of rigorous scientific data demonstrating improvement in symptoms compared with usual care; (2) the nearly universal lack of insurance coverage for medical cannabis therapies, resulting in a typical monthly cost to patients of $200–$300; and (3) concerns from patients and clinicians regarding potential side effects, the effect on current oncology treatments, and potential legal ramifications.

Researching patient response to cannabis is crucial, but the perspective of the clinician is also important. A large survey of medical oncologists estimated nearly 80% have had a discussion about cannabis with their patients at some point, and up to 46% have recommended cannabis use to a patient in the past year, however, details of potential barriers to actual use/implementation were not addressed.^10^ To assess oncology clinicians' views on medical cannabis (specifically regarding barriers to supporting and certifying patients) and to better ascertain the likelihood of reaching an acceptable enrollment census for our ongoing trial,^11^ we distributed a survey about medical cannabis to oncology clinicians practicing in Minnesota. Results were collected anonymously. The overall goal of the survey was threefold: (1) to assess current opinions and practice patterns regarding use of medical cannabis; (2) to identify barriers to medical cannabis use; and (3) to explore interest in future research and educational opportunities.

## Materials and Methods

### Study design and participants

From June to August 2017, we sent a 14-question survey to 552 oncology providers practicing in Minnesota, as identified through the Minnesota Board of Medical Practice database (for physicians who listed a hematology and/or oncology specialty in their Board application) and through an oncology NP database (for advanced practice nurses and PAs who care for adults and children with cancer in inpatient or outpatient settings). Participants who had an e-mail on file through the Board (*n*=266) were first e-mailed an invitation with a link to complete an online survey. Those who did not respond were sent as many as two e-mail reminders followed by a paper invitation and version of the survey. Clinicians without an e-mail address on file (*n*=286) were mailed a paper invitation and survey. Initial nonresponders were sent a second request 3 weeks later. Neither contingent nor noncontingent incentives were used. The survey and study were approved by the Institutional Review Board at HealthPartners Institute.

### Survey instrument

We designed the 14-item survey to identify providers' practices, knowledge, and attitudes about medical cannabis as well as to assess barriers to certifying patients in the MMCP (Supplementary Appendix S1). The survey was developed with multidisciplinary input from oncology clinicians, research staff, an oncology pharmacist, the manager of the MMCP, and a representative from each of Minnesota's cannabis manufacturers. Four questions sought demographic information (oncology provider role, years in practice, primary practice setting, and healthcare system affiliation). Two yes/no questions asked about providers' registration in the MMCP and whether they had certified any patients in the program. Two multiple-choice questions asked providers to indicate their confidence level in (1) discussing the risks and benefits of medical cannabis with patients who may be potential candidates and (2) explaining the MMCP to patients. One question inquired about whether informal or formal policies were in place at their organization to prevent or discourage enrollment in the MMCP. One question asked respondents to rate 10 different, preselected barriers on a 5-point Likert scale—ranging from 1 (no barrier) to 5 (very large barrier). One question assessed respondents' likelihood of offering our clinical study to eligible patients, and one question asked about what additional education the providers wanted regarding medical cannabis.

One item within the survey was previously published. In 2013, *The New England Journal of Medicine (NEJM)* described online the fictional case of a 68-year-old cancer patient who has metastatic breast cancer undergoing chemotherapy and is struggling with nausea, pain, and fatigue.^12^ Based on the information provided, the journal asked their readers to indicate whether they would recommend medical cannabis for that patient. With permission from the *NEJM*, we republished this scenario and poll and included it in our survey (Question No. 12) as an additional way to ascertain respondents' likelihood of prescribing medical cannabis to their patients.

### Data collection and management

Study data were collected and managed using REDCap (Research Electronic Data Capture) tools hosted at HealthPartners Institute.^13^ REDCap is a secure web-based application, compliant with the Health Insurance Portability and Accountability Act. Descriptive analyses were provided for each question and shown for all survey respondents (*n*=153) and then limited to registered respondents (*n*=68). Registered respondents were providers who stated that they had registered with MMCP and were thus eligible to certify patients for medical cannabis use. A mean Likert score was calculated for each potential barrier.

## Results

Of the 552 surveys distributed, 23 surveys did not reach the sender because of incorrect contact information. Excluding those potential respondents, 153 providers responded of the 529 surveys we distributed, corresponding to a response rate of 29%. We summarized results by analyzing two distinct groups: (1) all respondents (*n*=153) and (2) registered respondents (*n*=68). Results were analyzed separately for age and practice setting. However, these results showed no significant differences and thus are not presented.

### Demographics of survey respondents

Of the 153 participants who responded, the vast majority identified themselves as a medical oncologist or medical oncology NP/PA (*n*=125, 82%), and most practiced in a community setting (*n*=102, 67%; Table 1). Respondents represented a range of different hospitals and clinics across Minnesota, including nine of the largest healthcare systems headquartered in Minnesota, one system headquartered in an adjoining state but with a Minnesota presence, and the Minneapolis Veterans Administration Healthcare System. Just under half of respondents were registered with the MMCP (*n*=68, 44%), and 92% of registered respondents had certified a patient. Nearly all of the registered respondents were from medical oncology (96%).

**Table 1.** **Demographics of Providers with Completed Surveys**

Question^a^	Response	All respondents (*n*=153), *n* (%)	Registered respondents^b^ (*n*=68), *n* (%)
Role as an oncology provider	Medical oncologist	102 (67)	57 (84)
	Oncology NP/PA	23 (15)	8 (12)
	Radiation oncologist	21 (14)	None
	Other	7 (5)	3 (4)
Years in practice	0–5	20 (13)	7 (10)
	6–10	30 (20)	13 (19)
	11–15	21 (14)	12 (18)
	16–20	16 (10)	7 (10)
	21+	65 (42)	28 (41)
Practice setting	Academic (including VA)	50 (33)	15 (22)
	Community	102 (67)	50 (74)
Registered with MMCP	Yes	68 (44)	68 (100)
	No	82 (54)	N/A
Certified a patient in MMCP^c^	Yes	65 (42)	62 (92)
	No	88 (58)	6 (8)

^a^ Not all respondents answered all questions; thus, percentages may not add up to 100.

^b^ Includes only patients who stated they are registered with MMCP.

^c^ Three respondents claimed they certified a patient but were not registered in MMCP.

MMCP, Minnesota Medical Cannabis Program; NP, nurse practitioner; PA, physician assistant; VA, Veterans Affairs.

### Interest and ability to recommend cannabis

Among all survey respondents, 65% recommended use of medical cannabis for the *NEJM* patient scenario, 25% recommended against its use, and 10% left the question blank. These results were similar to those found by *NEJM* in which 76% of readers recommended use of medical cannabis and 23% did not.^14^ Registered respondents were more likely to recommend cannabis in the *NEJM* case scenario, with 85% recommending it.

Of the 153 respondents who commented on their organization allowing registration/certification in the MMCP, 104 (68%) noted that they do allow certification, 19 (12%) said they do not, and 30 (20%) were unsure.

### Barriers to discussing and offering cannabis certification

**FIG. 1.**
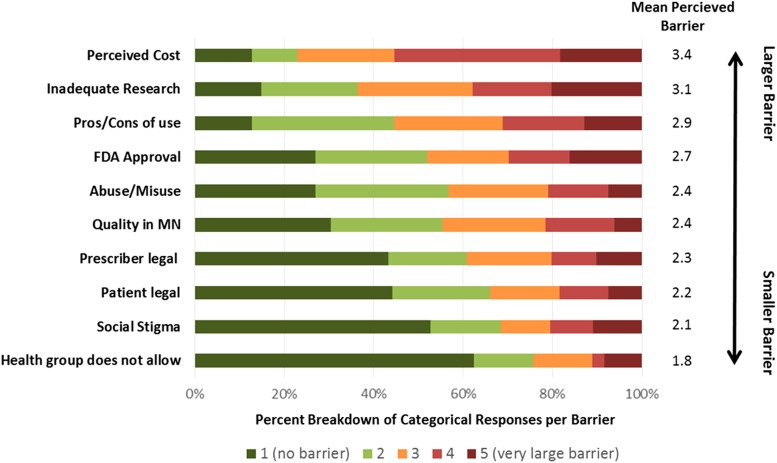
Barriers to discussing medical cannabis with qualified patients: average rating score and percent of responses to each response scale (1–5). FDA, Food and Drug Administration; MN, Minnesota.

Respondents reported a variety of barriers to discussing medical cannabis with qualified patients (Fig. 1). In the survey question, respondents rated 10 barriers on a scale of 1 (no barrier) to 5 (very large barrier). From largest to smallest barrier (with mean score from all respondents), responses were as follows: perceived cost to patient (3.4); research inadequate to justify use (3.1); unsure of side effects/benefits (pros/cons of use; 2.9); products are not Food and Drug Administration approved (2.7); concern about abuse/misuse (2.4); unsure of quality of products offered in Minnesota (quality in MN; 2.4); unsure of legal ramifications to me (provider legal; 2.3); unsure of legal ramifications to patient (patient legal; 2.2); I don't want to be identified as someone who prescribes medical cannabis (social stigma; 2.1); and my health group/leadership does not allow/support providers certifying patients (1.8).

### Engagement in medical cannabis 

#### education and research

We further assessed respondents' engagement in medical cannabis education and research through their responses to three questions in our survey (Table 2).

**Table 2.** **Engagement in Medical Cannabis Prescribing, Education, and Research**

Question^a^	Response	All respondents (*n*=153), *n* (%)	Registered respondents^b^ (*n*=68), *n* (%)
Confidence in discussing risks and benefits of medical cannabis	Very confident	34 (22)	26 (38)
	Somewhat confident	62 (41)	33 (49)
	Somewhat not confident	25 (16)	8 (12)
	Not at all confident	31 (20)	None
Interest in types of cannabis education^c^	Written summary	98 (64)	46 (68)
	Online learning program	66 (43)	31 (46)
	Symposia/conference	41 (27)	21 (31)
	Newsletter	24 (16)	13 (19)
	Other	4 (3)	4 (6)
	Not interested in more information	23 (15)	5 (7)
Likelihood of offering a randomized observational study with medical cannabis at no cost to the patient	Very likely	69 (45)	38 (56)
	Somewhat likely	49 (32)	20 (29)
	Somewhat unlikely	10 (7)	3 (4)
	Very unlikely	11 (7)	1 (1)

^a^ Not all respondents answered all questions; thus, percentages may not add up to 100.

^b^ Includes only patients who stated they are registered with MMCP.

^c^ Respondents could mark interest in more than one type of cannabis education. Of note, all respondents who marked “not interested in more information” selected only this option.

#### Confidence about discussing medical cannabis risks and benefits

A substantial number of respondents said they lacked confidence to discuss the risks and benefits of medical cannabis. About 36% of 152 respondents said they were “not at all confident” or “somewhat not confident” discussing the risks and benefits. The registered respondents had greater confidence overall, with only 12% stating they were “somewhat not confident.”

#### Interest in types of cannabis education

We asked participants what additional education they wanted regarding medical cannabis. Respondents could check one or more items to indicate that they were interested. Most expressed interest in getting additional training/education regarding cannabis. Rates were similar between all respondents and registered respondents. Of the 130 (85% overall) who wanted more training, 98 (75%) indicated interest in a written summary, 66 (51%) wanted an online learning program, 41 (32%) wanted a symposium or conference, and 24 (18%) wanted a newsletter.

#### Likelihood of offering clinical trial opportunities with medical cannabis to patients

Of the 148 respondents who indicated their likelihood of offering patients participation in our clinical trial assessing the impact of cannabis on opioid use and symptoms in patients with stage IV cancers, 45% and 32% said they were “very likely” or “somewhat likely,” respectively, to offer the study to patients. Among registered respondents, 85% said they were “very likely” or “somewhat likely” to offer the study.

## Discussion

This statewide survey of 153 oncology care providers in Minnesota provides important insight into current registration and certification patterns, identifies cost to patients and inadequate research as key clinician-identified barriers to medical cannabis use, and highlights the receptiveness of many providers to additional education and research to help them best provide information about medical cannabis to their cancer patients. Medical oncologists are the main providers registering with the MMCP and certifying cancer patients for use of cannabis, and they are overall highly supportive of our current randomized observational study with medical cannabis.^11^

In a 1990 survey of more than 1000 oncologists on the use of cannabis for chemotherapy-induced nausea/vomiting, “almost one half (48%) would prescribe marijuana to some of their patients if it were legal.”^15^ In a recent survey, more than 90% of pediatric oncologists expressed willingness to help children access medical cannabis.^16^ A sample of general healthcare practitioners showed that 83% of respondents support the use of medical cannabis for cancer patients with poorly controlled symptoms, and another recent survey of medical oncologists shows high levels of discussion occurring with patients and support for cannabis use by oncologists.^10,17^ Our survey showed that most adult oncology care providers continue to support cannabis by stating they would recommend it in the *NEJM* case scenario. Sixty-eight respondents identified as medical oncologists were registered with the MMCP (about half of our total survey respondents) at the time of survey completion. According to an *ad hoc* analysis conducted by the Minnesota Office of Medical Cannabis, as of December 21, 2017, 109 medical oncologists had registered in the program and, of these, 97 had certified at least one patient for a cancer-related condition. Approximately 302 active hematology/oncology physicians are practicing in Minnesota; thus, about a third have certified a patient to date.^18^

Although there is growing support for cannabis use in the cancer setting, significant barriers remain unaddressed. A separate survey of pediatric oncologists listed the following concerns about medical cannabis use: (1) absence of standards around cannabis dosing (46% of respondents); (2) children abusing medical cannabis (37%); and (3) fear of being prosecuted by the federal government (20%).^16^ Our results indicate that perceived cost to patients for medical cannabis use was the largest barrier to discussing medical cannabis with qualified patients. More than 50% of respondents stated this was a large barrier (rated 4 or 5 on a 1 to 5 scale).

In February 2017, we surveyed 320 patients in our clinic and found that 47% indicated cost as a barrier to use. In Minnesota, patients pay an annual $50–200 registration fee to be in the MMCP, and they typically pay anywhere from $100 to $300 per month (depending on patterns of use) to obtain cannabis from the two licensed manufacturers, who set the prices. These costs translate to an out-of-pocket expense of $3,000 to $3,500 per year for patients who use medical cannabis. These estimates are similar to those from chronic pain patients in New England who reported annual average spending for cannabis of $3,064 (median spending was $2,320).^19^

Respondents listed “my health group/leadership does not allow/support providers certifying patients” as the lowest overall barrier to discussing cannabis with their patients. However, about 24% of respondents felt it was a barrier (rating 3, 4, or 5). Of the 19 respondents who said that their primary organization does not allow registration/certification, 17 said that a formal policy precludes registration/certification (5 of those 17 were from the Minneapolis VA, a federal institution that does not allow cannabis use), while two said an informal policy prevented it. Those who noted that the primary organization did not allow registration/certification represented five different healthcare organizations. Even though this was the lowest overall barrier among all respondents, this could be a potentially large barrier in some systems, affecting numerous providers and patients alike.

Inadequate research into the benefits and risks of medical cannabis is seen as both a barrier and an opportunity. A number of cannabis products (with differing amounts of THC/CBD) have been studied in patients and assessed symptoms such as pain, nausea, and appetite loss.^20–26^ A few of the largest, randomized controlled trials (RCTs) using nabiximols focused mainly on pain and showed somewhat mixed results.^27,28^ A sentinel article from 1980 showed that 80% of patients prefer THC over prochlorperazine when used for chemotherapy-induced nausea.^5^ Subsequent small RCTs in the current antiemetic era have also shown promising results.^29,30^

Of note, respondents did not appear to be concerned with the current safety of medical cannabis, as seen through its status as a minor barrier. Data from the Minnesota Department of Health (MDH) support this finding. According to the MDH's report on the first year of the MMCP, from July 2015 to June 2016, “around 20 to 25% of enrolled patients report negative physical or mental side effects of some kind…the vast majority of adverse side effects, around 90%, are mild to moderate in severity.”^2^

Currently, medical oncologists get little to no formal training in medical school, residency, or fellowship on medical cannabis. However, support for ongoing education was high in our survey, with only 15% of respondents indicating that they were not interested in receiving further education. Furthermore, pharmacists in Minnesota also desire further training and education.^31^ In Minnesota, oncology providers only certify that a patient has a medical condition that allows them access to cannabis, while the cannabis distribution center pharmacist recommends the doses/types of cannabis and monitors for dose modifications and side effects long term. With more states legalizing medical/recreational use, patients are requesting information about cannabis from their cancer team, yet only 15% report receiving it.^3^ One reason for this lack of dialogue may be due to providers' lack of comfort with their own knowledge. A previous healthcare provider survey conducted in Washington State revealed a low level of self-reported knowledge and comfort in recommending medical cannabis.^32^ Clinicians lack of solid knowledge may come from many factors, including (1) difficulty tracking the many different types of cannabis programs/requirements (e.g., state laws on recreational vs medical program), (2) the fact that cannabis remains a schedule one drug and, consequently, clinicians have little direct experience overseeing its use, and (3) the number of large, high-quality randomized studies showing both efficacy and safety of cannabis in the cancer setting is limited. While the respondents of this broader discipline survey agreed that clinicians should have structured training and education on medical cannabis, less than a quarter (24%) received information about medical cannabis through lectures or continuing medical education programming. Education seemed to have a clear effect on engagement in medical cannabis prescribing; clinicians who had written authorizations for medical cannabis were more likely to have received training than those who did not write authorizations.^32^

Studies conducted among clinicians in Colorado^33^ and Canada^34^ have yielded findings similar to that of the Washington State survey.^32^ Through this previous research, two clear takeaways emerge: the primary source of surveyed clinicians' source of information is informal channels (e.g., the media, other clinicians, and patients), however, if training were available, clinicians would participate. While our Minnesota survey did not inquire about where respondents were receiving information on medical cannabis, our participants expressed strong interest in receiving additional education about medical cannabis and our state's medical cannabis program. Educating clinicians with reliable, objective information on cannabis is paramount to facilitate improved discussions with patients about benefits, risks, and cost considerations. We plan to use open-ended feedback responses from this survey to conduct focus groups with clinicians and leadership at the MDH to improve educational opportunities regarding cannabis use in the oncology setting.

This study had several limitations. The sample size was small and may not be generalizable to other states. Our response rate was lower than other studies. One reason for the low response rate could be because we did not provide cash incentives for survey completion. While all major health systems throughout Minnesota were well represented through survey participation, we were not able to extrapolate the survey results in a statistically significant way across the Minnesota healthcare landscape. Response bias, implicit in self-selected survey enrollment, presented another inherent limitation for this study; participants could have a disproportionate level of awareness and motivation around the topic of medical cannabis programs and participation. Such response bias could be reflected in participants' overwhelming support for our study and the high support for ongoing education. Of note, however, were respondents' gaps in knowledge about medical cannabis and the MMCP, which could be readily discerned through their responses, particularly the open-ended ones. Moreover, a case could be made that those who are decidedly opposed to cannabis might be more likely to voice their opinions in this survey. That said, ultimately, our study results afforded little opportunity to compare responders and nonresponders. Finally, the *NEJM* scenario and the research opportunity questions offer limited answer choices and focus on specific indications for cannabis. For example, the trial opportunity discussed only pertained to patients with stage IV cancers requiring opioids. Thus, our survey may not have allowed respondents to give more detailed answers for use of cannabis in a wide variety of situations.

## Conclusion

Our survey of oncology providers in Minnesota assessed current practice patterns, identified barriers to discussing cannabis with qualified patients, and explored opportunities for future research and education. There is a clear need for well-conducted clinical trials to provide reliable data to guide clinicians in their discussions about the benefits, risks, and cost considerations of using cannabis to help control cancer-related symptoms. With the results of this survey and our ongoing clinical trial, we hope to better understand what role medical cannabis may have in the care of patients with cancer.

## Supplementary Material

Supplemental data

## Abbreviations Used

APRN: advanced practice registered nurse
CBD: cannabidiol
FDA: Food and Drug Administration
MDH: Minnesota Department of Health
MMCP: Minnesota Medical Cannabis Program
NEJM: The New England Journal of Medicine
NPs: nurse practitioners
PA: physician assistant
RCTs: randomized controlled trials
THC: tetrahydrocannabinol
