# Supplementary material for: Oncology Clinicians and the Minnesota Medical Cannabis Program: A Survey on Medical Cannabis Practice Patterns, Barriers to Enrollment, and Educational Needs
Source: Cannabis Cannabinoid Res. 2018 Oct 1;3(1):195–202. doi: 10.1089/can.2018.0029 (PMC6225592; doi:10.1089/can.2018.0029)
Supplement: Supplemental data [file Supp_Appendix1.pdf]

HealthPartners Institute

Medical Cannabis Oncology Provider Survey

Our team would like to better understand the opinions of oncology care providers about the use of medical cannabis. Your results will help us as we design future research studies. Please help us by completing this survey. Your responses are confidential and will only be reported in aggregate.

1. What is your role as an oncology provider?

- ◯ Medical Oncologist
- ◯ Oncology Advanced Practice Clinician (NP, PA)
- ◯ Radiation Oncologist
- ◯ Surgeon
- ◯ Palliative Care Clinician
- ◯ Other (please specify): ______________________

2. How many years have you been practicing oncology?

- ◯ 0 to 5 years
- ◯ 6 to 10 years
- ◯ 11 to 15 years
- ◯ 16 to 20 years
- ◯ 21+ years

3. How would you describe your primary practice setting?

- ◯ Academic
- ◯ Community
- ◯ Other (please specify): ______________________

4. What is your primary healthcare system affiliation?

- ◯ Affiliated Community Medical Centers
- ◯ Allina
- ◯ CentraCare
- ◯ Children's Hospitals and Clinics
- ◯ Essentia
- ◯ Fairview
- ◯ Gillette Children's Hospital
- ◯ HealthPartners
- ◯ HealthEast
- ◯ Hennepin County Medical Center
- ◯ Mayo
- ◯ North Memorial Health
- ◯ Olmsted Medical Center
- ◯ Park Nicollet
- ◯ Sanford Health
- ◯ St. Luke's
- ◯ University of Minnesota
- ◯ VA Health Care System
- ◯ Other (please specify): ____________________

5. Have you registered with the Minnesota Medical Cannabis Program in order to certify patients?

- ◯ Yes
- ◯ No

6. Have you certified any patients for the Minnesota Medical Cannabis Program?

- ◯ Yes
- ◯ No

7. How confident are you in your ability to discuss the risks and benefits of medical cannabis with a patient who may be a candidate for certification?

- ◯ Very confident
- ◯ Somewhat confident
- ◯ Somewhat not confident
- ◯ Not at all confident

8. How confident are you in your ability to describe to patients the organization and operations of the Minnesota Medical Cannabis Program? (e.g., certifiable conditions, process for patient participation, role of medical cannabis distribution centers, etc.)

- ◯ Very confident
- ◯ Somewhat confident
- ◯ Somewhat not confident
- ◯ Not at all confident

9. Does your primary organization allow you to register and enroll patients in the Minnesota Medical Cannabis Program?

- ◯ Yes
- ◯ No, there is a formal policy that prevents this
- ◯ No, there is an informal policy that prevents this
- ◯ Unsure

10. How much of a barrier do you consider each of the following reasons to be in discussing medical cannabis with qualified patients?

	No barrier 1	2	3	4	Very large barrier 5
a. Perceived cost to patient	◯	◯	◯	◯	◯
b. Unsure of side effects/benefits	◯	◯	◯	◯	◯
c. Research inadequate to justify use	◯	◯	◯	◯	◯
d. Unsure of quality of products offered in Minnesota	◯	◯	◯	◯	◯
e. Products are not FDA approved	◯	◯	◯	◯	◯
f. Unsure of legal ramifications to me	◯	◯	◯	◯	◯
g. Unsure of legal ramifications to patient	◯	◯	◯	◯	◯
h. Concern about abuse/misuse	◯	◯	◯	◯	◯
i. I don't want to be identified as someone who prescribes medical cannabis	◯	◯	◯	◯	◯
j. My health group/leadership does not allow/ support providers certifying patients	◯	◯	◯	◯	◯

k. Other (please elaborate): ______________________________

11. **In efforts to examine the effectiveness of medical cannabis on cancer-related symptoms, we are currently designing a research study for patients with stage IV cancers undergoing chemotherapy and requiring opioids. In this study, half of the patients would be randomly assigned to receive medical cannabis immediately for 3 months at no cost. The other half of the patients would receive medical cannabis 3 months later (also at no cost and for a 3 month duration). All patients would complete a monthly symptom survey over the phone.**

    If you had an eligible patient, how likely would you be to offer this study to them?

    - ◯ Very likely
    - ◯ Somewhat likely
    - ◯ Somewhat unlikely
    - ◯ Very unlikely
    - ◯ Other (please elaborate): ____________________

    ____

12. ***In 2013, the NEJM published this online scenario:***

    Marilyn is a 68-year-old woman with metastatic breast cancer metastatic to the lungs and bones. She is currently undergoing chemotherapy with doxorubicin. She reports having very low energy, minimal appetite, and substantial pain in her thoracic and lumbar spine. For relief of nausea, she has taken ondansetron and prochlorperazine, with minimal success. She has been taking 1000 mg of acetaminophen every 8 hours for the pain. Sometimes at night she takes 5 mg or 10 mg of oxycodone to help provide pain relief. During a visit with you she asks about the possibility of using marijuana to help alleviate the nausea, pain, and fatigue.

    As her physician, which one of the following approaches do you find appropriate for this patient? Base your choice on the published literature, your clinical experience, recent guidelines, and other sources of information.

    - ◯ Recommend the Medicinal Use of Marijuana
    - ◯ Recommend against the Medicinal Use of Marijuana

13. **What additional education regarding medical cannabis would you be interested in receiving? Please mark all educational forms that would interest you.**

    - ◯ Written summary of peer-reviewed literature
    - ◯ Online learning programs (e.g., video lectures, powerpoint slides)
    - ◯ Symposia/conferences
    - ◯ Newsletter
    - ◯ I am not interested in receiving further education
    - ◯ Other (please elaborate): ____________________

    ____

    ____

14. Please provide any other comments you have on the use of medical cannabis for patients with cancer.

Thank you for your time!

Dylan Zylla, MD, MS
Co-Medical Director of Oncology Research (Park Nicollet)
Adjunct Assistant Professor (University of Minnesota)
Dylan.Zylla@parknicollet.com
